# Very low prevalence of haemosporidian parasites in two species of marsh terns

**DOI:** 10.1007/s00436-023-07997-y

**Published:** 2023-11-01

**Authors:** Anna Dubiec, Natalia Atamas, Mateusz Ledwoń

**Affiliations:** 1 Museum and Institute of Zoology, Polish Academy of Sciences, Wilcza 64, 00-679 Warsaw, Poland; 2 Department of Animal Monitoring and Conservation, Laboratory of Population Ecology, I. I. Schmalhausen Institute of Zoology, National Academy of Sciences of Ukraine, B. Khmelnytskoho Str., 15, Kyiv, 01601 Ukraine; 3 Institute of Systematics and Evolution of Animals, Polish Academy of Sciences, Sławkowska 17, 31-016 Cracow, Poland

**Keywords:** Black Tern, Haemosporidian parasites, Laridae, Migratory birds, Whiskered Tern

## Abstract

Vector-transmitted haemosporidians are among the most common parasites in birds, but our knowledge of the inter-specific patterns of infection rates and the parasite community composition is far from complete because of the unequal distribution of the screening effort across bird families and genera. To assess infection rates and the diversity of haemosporidians from the genera *Plasmodium*, *Haemoproteus*, and *Leucocytozoon* in marsh terns, which represent poorly explored in this regard genus of the family gulls, terns, and skimmers (Laridae), we screened two species: the Whiskered Tern (*Chlidonias hybrida*) and the Black Tern (*Chlidonias niger*). We sampled these long-distance migratory birds on breeding grounds: the Whiskered Tern in south-central Poland and north-central Ukraine, and the Black Tern—in north-central Ukraine. We found that birds from both species were infected only sporadically, with prevalence at the population level not exceeding 3.4%. Only parasites from the genera *Plasmodium* and *Leucocytozoon* were detected. There was neither an inter-specific difference nor a difference between populations of the Whiskered Tern in infection rates. In total, we registered three lineages—one *Plasmodium* and two *Leucocytozoon*—that were previously recorded in other bird species, and two unidentified *Plasmodium* infections. One of the lineages (*Leucocytozoon* LARCAC02) represents a specialist parasite with the host range restricted to larids and geographic range restricted to Poland, and two others (*Plasmodium* SGS1 and *Leucocytozoon* CIAE02) represent generalist parasites with very broad host and geographic ranges. This study reinforces the existing evidence that terns host parasites from genera *Haemoproteus*, *Plasmodium*, and *Leucocytozoon* only sporadically.

## Introduction

Parasitic protozoa from genera *Plasmodium*, *Haemoproteus*, and *Leucocytozoon* (phylum: Apicomplexa, order: Haemosporida) are among the most common parasites of birds (Atkinson and van Riper III 1991; Valkiūnas 2005). They cause avian malaria and malaria-like diseases associated with effects ranging from relatively benign in populations with endemic infections to severe in naive populations and non-indigenous birds (e.g. Atkinson and Samuel 2010; Podmokła et al. 2017). To complete the life cycle, haemosporidian parasites require arthropod hosts, which act as their vectors and vertebrate hosts. Numerous bloodsucking arthropod species from several families may transmit haemosporidian parasites including hippoboscid flies (Hippoboscidae), biting midges (Ceratopogonidae), mosquitoes (Culicidae), and blackflies (Simuliidae) (Santiago-Alarcon et al. 2012). The distribution of haemosporidian parasites across avian taxa is very uneven ranging from very high infection rates, e.g. in some Paridae and Columbidae species, to very low rates or complete lack of infection in families such as Caprimulgidae and Scolopacidae (Bennett 1993; Bensch et al. 2009). Representatives of most bird orders have been tested to date for haemosporidian parasites; however, due to an unequal distribution of the screening effort across bird families and genera, our knowledge of the inter-specific differences in infection rates and the parasite community is far from complete (Valkiūnas 2005; Bensch et al. 2009).

Gulls, terns, and skimmers (Laridae) are a family of 99 species classified in 23 genera (Clements et al. 2022). Most larid species inhabit coastal areas, but some of them are pelagic for most of the year, while others occupy inland habitats, in most cases aquatic (Winkler et al. 2020). Available data places Laridae among the families on the low spectrum of infection rates with haemosporidians, although there are some exceptions. Based on the data from 36 larid species, Quillfeldt et al. (2011) reported the mean prevalence of haemoparasitic infections (*Plasmodium*, *Haemoproteus*, *Leucocytozoon*, *Hepatozoon*, and *Babesia*) in this family at the level of 9.2%. However, despite the overall low prevalence infection rates in some larid species may reach very high levels. For example, infection rates of 94% in Caspian Gulls (*Larus cachinnans*) and 100% in Herring Gulls (*Larus argentatus*), primarily caused by *Leucocytozoon* parasites, were reported in a mixed colony occupying freshwater habitat in central Poland (Zagalska-Neubauer and Bensch 2016). A high prevalence (89–92%) was also found in some colonies of Yellow-legged Gulls (*Larus michahellis*) and Audouin’s Gulls (*Ichthyaetus audouinii*) on Mediterranean islands (Ruiz et al. 1995; Bosch et al. 1997), and recently in the Black-headed Gull (*Chroicocephalus ridibundus*), in which infection rates with *Plasmodium*/*Haemoproteus* ranged between 35 and 75% among seven colonies located across Poland (Włodarczyk et al. 2022). However, while birds from some genera of Laridae family have been rather well screened, other genera are poorly represented in the literature on haemosporidian infections. One such genus is *Chlidonias*, i.e. marsh terns, represented by four species: the Whiskered Tern (*Chlidonias hybrida*), the Black Tern (*Chlidonias niger*), the White-winged Tern (*Chlidonias leucopterus*), and the Black-fronted Tern (*Chlidonias albostriatus*) (Clements et al. 2022).

Here, we used blood samples from two species of marsh terns, the Whiskered Tern and the Black Tern, to screen for the presence of parasites from genera *Plasmodium*, *Haemoproteus*, and *Leucocytozoon.* The Whiskered and Black Terns are colonial, medium-sized, socially monogamous birds, with moderate sexual size dimorphism (Cramp 1985). The Whiskered Tern breeds across large areas of the Palearctic, Africa, and Australia, while the Black Tern breeds in Western Palearctic and North America (Cramp 1985). Both species build nests on water plants located on inland marshes, fish ponds, dam reservoirs, oxbows, and river valleys (Cramp 1985; Ledwoń et al. 2014; Goławski et al. 2015). They breed both in mono-specific colonies and in colonies with other species—gulls (Larinae), terns (Sterninae), or grebes (Podicipedidae) (Ledwoń et al. 2014). Both species are migratory except for some populations of the Whiskered Tern in South Africa and Asia. Eurasian Black Terns winter along the coasts of West Africa, from Mauritania in the north to Namibia in the south (Cramp 1985; van der Winden et al. 2014). At nonbreeding grounds in West Africa, they spend substantial time far offshore (van der Winden et al. 2014). They also may stay in coastal areas and freshwater lakes, predominantly near coasts but they were occasionally reported also at inland lakes (Winkler et al. 2020). Whiskered Terns from Eurasia winter mainly in West and East Africa, at freshwater wetlands both inland and on coasts (Winkler et al. 2020). An exceptionally large assemblage of Whiskered Terns has been recorded in the Nile delta region (Cramp 1985).

Currently, data on the occurrence of haemosporidians in marsh terns are very scarce and limited to blood smear screening. Greiner et al. (1975) reported no haemosporidians in two screened individuals of the Black Tern and Peirce (1981), after Labbé (1984), reported no haemosporidians in the unknown number of screened individuals of the same species. Similarly, Figuerola (1999), after Bennett et al. (1982) and Bishop and Bennett (1992), reported no haemoparasites in the unknown number of White-winged Terns and Black Terns. Also terns from other genera, including sister genera *Sterna* (typical black-capped terns) and *Thalasseus* (crested terns), were shown to be free of haemosporidian parasites or hold very low infection rates in most screened wild populations (Table 1). The only exceptions are the Forster’s Tern, the Gull-billed Tern, and the Large-billed Tern with 25%, 30%, and 16.2% prevalence of haemosporidian infections, respectively (Greiner et al. 1975; Roos 2014; Fecchio et al. 2021). However, in Forster’s Terns and Gull-billed Terns, prevalence estimates are associated with high uncertainty since they were based on small sample sizes (Jovani and Tella 2006). In the Large-billed Tern, only juveniles and nestlings were sampled (Roos 2014). Infection rates commonly differ between nestlings/juveniles and adult birds with adults being more prevalently infected (Wojczulanis-Jakubas et al. 2012; Garcia-Longoria et al. 2015, but see Hammers et al. 2016). Therefore, inter-specific comparisons of infection rates based on birds from different age groups are not reliable.

**Table 1:** The literature survey on the occurrence of haemosporidian parasites (genera *Plasmodium*, *Haemoproteus*, *Leucocytozoon*) in terns from wild populations. Sampling location, prevalence (Prev), number of infected individuals (Ninf), sample size (Nall), and the reference are presented. Studies in which hosts were screened molecularly are marked with an asterisk, and the study which used both methods is marked with a double asterisk (in column Prev). All other studies were based on blood smear screening. The genus of detected parasites is given in column Ninf as P—*Plasmodium*, H—*Haemoproteus*, and L—*Leucocytozoon*. If the host age category was specified, it is presented in the parentheses in the column Nall with chicks coded as ch, juveniles as juv, and adults as ad. The taxonomy of host species follows Clements et al. (2022)

Species	Sampling location	Prev	Ninf	Nall	References
Common Tern (*Sterna hirundo*)	North America	0.0	0	587	Greiner et al. (1975)
	India	0.0	0	2	Mandal et al. (1989)
	Bird Is., Plover Is., New Is., USA	0.0	0	75 (ad)	Fiorello et al. (2009)
	Poland	1.2*	2 (L)	161 (ad)	Włodarczyk et al. (2022)
	Germany	0.0*	0	322 (ad)	Włodarczyk et al. (2022)
	Argentina	3.6**	2 (P)	56 (ad)	Capasso et al. (2023)
Roseate Tern (*Sterna dougallii*)	North America	0.0	0	30	Greiner et al. (1975)
Forster’s Tern (*Sterna forsteri*)	Iowa, USA	25.0	1 (P)	4 (ad)	Coatney (1938)
Arctic Tern (*Sterna paradisaea*)	North America	0.0	0	20	Greiner et al. (1975)
River Tern (*Sterna aurantia*)	India	0.0	0	1	Mandal et al. (1989)
Little Tern (*Sternula albifrons*)	North America	0.0	0	1	Greiner et al. (1975)
	India	0.0	0	1	Mandal et al. (1989)
Sandwich Tern (*Thalasseus sandvicensis*)	Western Europe	0.0	0	1	Peirce 1981
Cayenne Tern (*Thalasseus eurygnathus*)	Patagonia, Argentina	0.0	0	17 (11 ad + 6 ch)	Jovani et al. (2001)
Royal Tern (*Thalasseus maximus*)	Patagonia, Argentina	0.0	0	21 (ch)	Jovani et al. (2001)
Great Crested Tern (*Thalasseus bergii*)	Phillip and Montague Is., Australia	0.0	0	243 (ch)	Watson (2012)
	Java, Indonesia	0.0*	0	4 (ad)	Yuda (2019)
Lesser Crested Tern (*Thalasseus bengalensis)*	India	0.0	0	1	Mandal et al. (1989)
Gull-billed Tern (*Gelochelidon nilotica*)	North America	0.0	0	1	Greiner et al. (1975)
	India	0.0	0	1	Mandal et al. (1989)
	Australia	30.0*	3 (H)	10	Fecchio et al. (2021)
Sooty Tern (*Onychoprion fuscatus*)	Johnston Atoll, USA	0.0	0	72 (37 ad + 35 ch)	Work (1996)
	Offshore islands of Northern Mexico	0.0	0	50 (ad + juv)	Clark and Swinehart (1969)
	Bird Is., Seychelles	0.0	0	3 (ch)	Peirce and Feare (1978)
	Goelette Is., Seychelles	0.0	0	4 (ch)	Peirce and Feare (1978)
	Desnoeufs Is., Seychelles	0.0	0	17 (ch)	Peirce and Feare (1978)
	Acadia Island, Pitcairn Islands	0.0	0	6 (ch)	Peirce and Brooke (1993)
Bridled Tern (*Onychoprion anaethetus*)	Penguin Is., Australia	0.0	0	51 (ad)	Campbell (2015)
Large-billed Tern (*Phaetusa simplex*)	Amazon basin, Brazil	16.2	12 (P/H)	74 (2 ch + 72 juv)	Roos (2014)
Black Noddy (*Anous minutus*)	Offshore islands, Brazil	0.0	0	42 (34 ad + 8 juv)	Quillfeldt et al. (2014)
Brown Noddy (*Anous stolidus*)	Aldabra Atoll, Seychelles	4.2	1 (H)	24 (ad)	Lowery (1971)
	Oeno Atoll, Pitcairn Islands	0.0	0	1	Peirce and Brooke (1993)
	Offshore islands of Northern Mexico	0.0	0	2	Clark and Swinehart (1969)
	Bird Is., Seychelles	0.0	0	1 (ad)	Peirce and Feare (1978)
	Tutuila, American Samoa	0.0	0	1	Atkinson et al. (2006)
	Offshore islands, Brazil	8.2* 0*	8 0	98 (ad) 73 (juv)	Quillfeldt et al. (2014)

Our primary aim was to assess the prevalence of infection and the community composition of haemosporidian parasites in Whiskered and Black Terns. We expected that the prevalence of haemosporidian infections in both species would be very low. We based this expectation on the fact that terns from sister genera *Sterna* and *Thalasseus* are either not infected or infected sporadically, and host phylogenetic relationships have been shown to be related to the prevalence of haemosporidian parasites (Ellis et al. 2020). Moreover, we predicted that Whiskered Terns are either more prevalently infected or are infected with a more diverse haemosporidian community than Black Terns because some habitats occupied by the former species during the nonbreeding season should support a higher abundance of arthropods vectoring haemosporidians than marine and coastal habitats occupied by Black Terns (Valkiūnas 2005). Such differences have been, for example, found in some shorebirds with species, that occupy during the nonbreeding season freshwater inland habitats, being more prevalently infected than species from marine coastal habitats (Mendes et al. 2005; Yohannes et al. 2009). Whiskered Terns may be exposed to haemosporidian vectors not only on breeding but also on wintering grounds, while Black Terns—primarily on breeding grounds. Based on geolocator data, the Nile delta (primarily the vicinity of seaside lakes Burullus and Manzala) and the interior lakes Victoria and Chad, were identified as wintering sites of Whiskered Terns breeding in south-central Poland (M. Ledwoń, unpubl. data). The exact wintering sites of Whiskered Terns from north-central Ukraine are currently unknown. As for Black Terns from north-central Ukraine, the only recovery record linking wintering and breeding sites was from saltworks on the Atlantic coast in Namibia (N. Atamas, unpubl. data). This observation is in line with other ringing records which show that Eurasian Black Terns from more eastern breeding areas tend to winter in West Africa more southwards, along the coast of Namibia, than birds from more western breeding grounds (Haverschmidt 1978, Glutz von Blotzheim and Bauer 1982, as cited in Szczys et al. 2017). Because Whiskered Terns were sampled in two geographically distant populations (south-central Poland and north-central Ukraine), we also tested for inter-population variation in infection rates in this species. We had no specific predictions in this respect, but intra-specific differences among populations in haemosporidian infection rates are commonly reported (Sol et al. 2000; Włodarczyk et al. 2022). Birds were screened for the presence of haemosporidian parasites in the bloodstream using molecular methods, which in many studies have been found to be more sensitive than blood smear screening (Durrant et al. 2006; Garamszegi 2010, but see Valkiūnas et al. 2008), and which allow for the identification of genetic variants of parasites (Bensch et al. 2009).

## Methods

### Sampling sites and sample collection

**Fig. 1:**
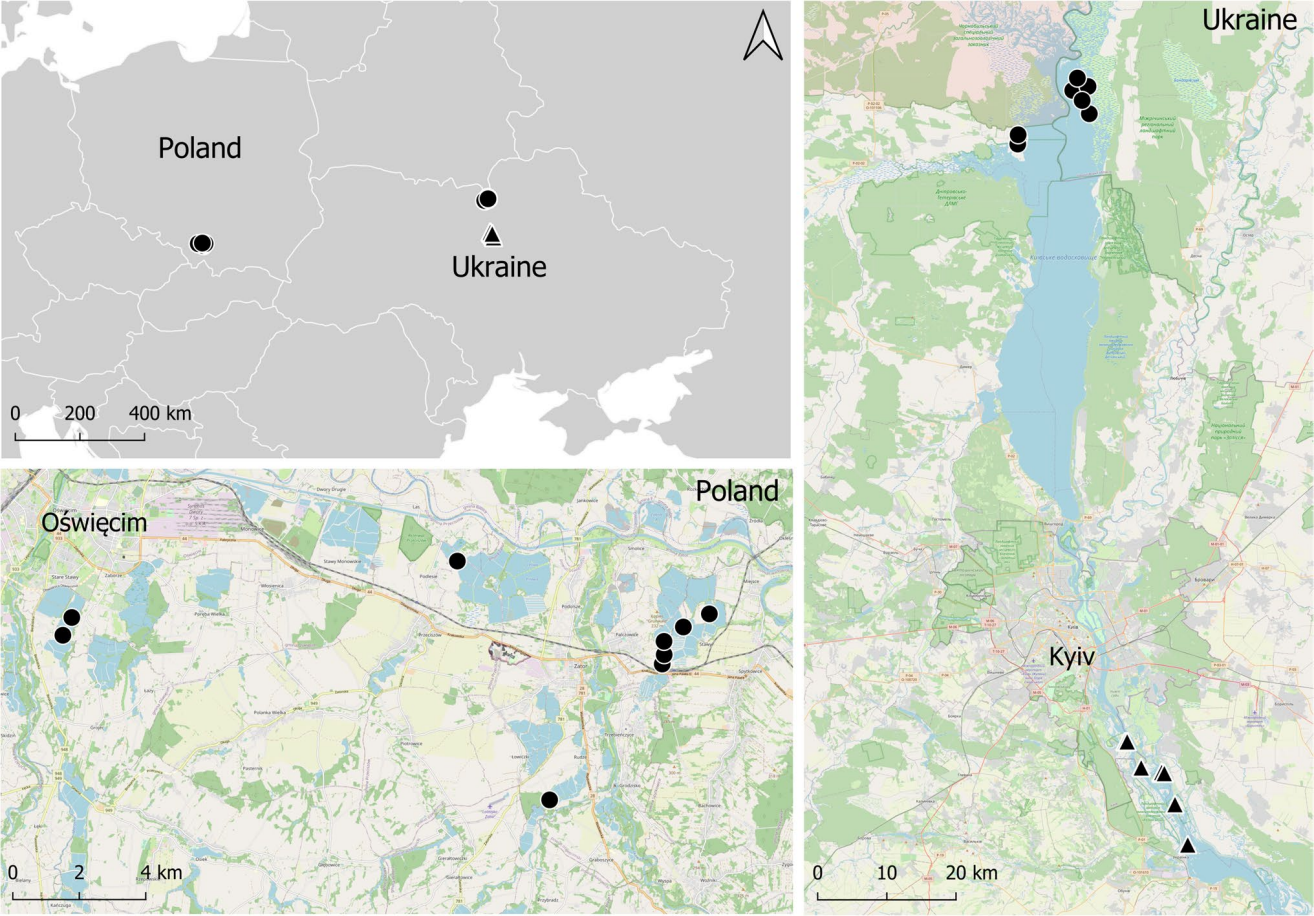
The location of sites where Whiskered and Black Terns were sampled for blood to screen for haemosporidian infections. Black circles represent sampling sites of Whiskered Terns and black triangles—sampling sites of Black Terns

We sampled Whiskered Terns for blood in 2013–2015 on a carp pond complex in the Upper Vistula valley in southern Poland and in 2015–2017 on a dam reservoir on the Dnipro River north of Kyiv in Ukraine and Black Terns—in 2016–2017 on a dam reservoir on the Dnipro River south of Kyiv (Fig. 1). Blood samples were collected within the framework of projects focusing primarily on the breeding biology of the study species (e.g. Ledwoń and Neubauer 2017; Atamas and Tomchenko 2020). Birds nested mainly on floating or emergent vegetation (e.g. Water Chestnuts (*Trapa natans*) and Fringed Water Lilies (*Nymphoides peltata*)) including tufts and mats of rotting and withered aquatic plants. They were caught at the nests with roof traps during incubation or chick’s rearing period between June and August. Approximately 0.2 ml of blood was collected from the tarsus vein using a needle and a heparinized capillary and stored in 98% ethanol. In total, 216 blood samples were used in the analyses of haemoparasitic infections: 87 and 46 samples from Whiskered Terns collected in Poland and Ukraine, respectively, and 83 samples from Black Terns in Ukraine.

### Molecular screening for blood parasites

Host and parasite DNA was extracted from blood samples using Blood Mini kit (A&A Biotechnology, Gdańsk, Poland). Birds were screened for the presence of *Haemoproteus*, *Plasmodium*, and *Leucocytozoon* with the multiplex PCR developed by Ciloglu et al. (2019). The protocol is based on the use of three primer sets that amplify DNA fragments of different sizes depending on the parasite genus. Specifically, PMF-PMR primers amplify in *Plasmodium* parasites a 377–379-bp long fragment of a non-coding region of the mtDNA, HMF-HMR primers amplify in *Haemoproteus* parasites a 525–533-bp long fragment between the 5′ end of cytochrome *b* and a non-coding region of mtDNA, and LMF-LMR primers amplify in *Leucocytozoon* parasites a 218-bp long fragment of the cytochrome c oxidase subunit 1 (COX1) gene of mtDNA. The use of this protocol allows for the identification of the parasite genus in a single PCR without the necessity of sequencing a product. Moreover, this multiplex protocol detects multiple infections at a higher rate than the commonly used nested PCR protocol by Hellgren et al. (2004). PCR reaction mix (2 × Qiagen Multiplex PCR Master Mix, Qiagen), as well as PCR thermal profile, followed Ciloglu et al. (2019). Each PCR run contained a negative control (ddH_2_O) for every 22 samples and one positive control. As a positive control, a DNA isolate from the Great Tit (*Parus major*) infected with parasites representing all three genera was used. Negative and positive controls were used to test for contamination and PCR failure, respectively. The PCR products (8.0 μl) were resolved on 2% agarose gel stained with SimplySafe (Eurx, Gdańsk, Poland) and visualised under UV light. All samples were screened twice (except for one sample with a limited volume, which provided a well-visible product in the first multiplex PCR run) to ensure the repeatability of results, and those samples that produced inconsistent results were tested the third time. The individual was scored as infected if the product was amplified in the two first replicates or two out of three PCR replicates in the case of isolates with inconsistent results.

Samples scored as positive with the multiplex PCR were next run with a nested PCR protocol developed by Hellgren et al. (2004) to identify parasites to the lineage level as listed in the MalAvi database (Bensch et al. 2009). The nested PCR targets an approximately 480-bp long fragment of the cytochrome *b* gene in parasites from the genera *Plasmodium*, *Haemoproteus*, and *Leucocytozoon*. In the first PCR, a primer pair HaemNFI–HaemNR3 amplifies the DNA fragment from all three haemosporidian genera. In the second step, in which the product from the first PCR is used as a template, in two separate PCRs either the product from *Haemoproteus* and *Plasmodium* parasites (primer pair HaemF–HaemR2) or *Leucocytozoon* parasites (primer pair HaemFL–HaemR2L) is amplified. The composition of the PCR mix followed Kubacka et al. (2019) and PCR thermal conditions—the original protocol, except for the duration of the initial denaturation which lasted 2 min instead of 3 min, as recommended by the manufacturer of the Taq polymerase (GoTaq G2 Hot Start polymerase, Promega). The plate contained also a negative and a positive control as described above. The amplification of the product was confirmed by running 6 μl of the PCR product on a 2% agarose gel stained with SimplySafe. We found that two *Leucocytozoon* lineages that we registered in terns in this study were also amplified with a pair of primers specific for *Haemoproteus* and *Plasmodium* parasites. These primers yielded a PCR product visible as a strong band on an agarose gel, and when sequenced bidirectionally, produced good-quality sequences only with a reverse primer (HaemR2). These sequences showed 100% concordance with sequences amplified from the same DNA isolates with primers specific for *Leucocytozoon*. Such non-specific co-amplification of *Leucocytozoon* lineages with primers HaemF–HaemR2 targeting *Haemoproteus* and *Plasmodium* parasites has already been described by Cosgrove et al. (2006) and has been reported to date in several other studies (Lewicki 2013; Capilla-Lasheras et al. 2017; Lynton-Jenkins et al. 2020).

All samples that amplified a product of an appropriate size in a nested PCR were cleaned enzymatically (Exo-Sap) and Sanger sequenced bi-directionally by an external company (Genomed, Warsaw, Poland). Chromatograms were inspected visually for the presence of multiple peaks, which indicate mixed infections. Sequences were trimmed and assembled into consensus sequences using BioEdit software ver. 7.2.3 (Hall 1999). Consensus sequences were compared with the sequences deposited in the MalAvi database using a built-in BLAST function (Bensch et al. 2009).

The quality of DNA isolates that were scored as negative was checked with the protocol for molecular sexing (see below). If a DNA isolate failed to produce a PCR product in two replicates, the sample was excluded from the estimates of infection rates (*n* = 2).

### Molecular sex identification

Birds were sexed by amplifying a fragment of a sex-linked chromo-helicase-DNA-binding (CHD) gene. To that end, a primer set designed by Fridolfsson and Ellegren (1999)—2550F and 2718R—was used with the following PCR thermal profile: initial denaturation at 94°C for 2 min, 35 cycles: denaturation at 94°C for 30 s, annealing at 50°C for 30 s, extension at 72°C for 1 min, and final extension at 72°C for 5 min. Ten μl of PCR reaction mix consisted of 0.2 mM dNTP, 1.5 mM MgCl_2_, 1 × PCR buffer, 0.5 μM of each primer, and 0.5 U of Taq DNA polymerase (GoTaq G2 Hot Start Polymerase, Promega, USA). Amplicons were resolved on 3% agarose gel and visualised under UV light. Samples that produced two bands were scored as females and which produced one band as males.

### Phylogenetic analyses

To examine phylogenetic relationships between the two *Leucocytozoon* lineages recovered from marsh terns in this study and the set of closely related lineages (lineages with 98.95% similarity in the case of CIAE02 and 97.27% similarity in the case of LARCAC02 as assessed with BLAST in GenBank accessed on February 3, 2023) as well as selected sequences matched to *Leucocytozoon* morphospecies, the Bayesian inference was used. In total, the dataset contained 49 sequences. All sequences were 476 bp long, without gaps and unambiguous sites. *Leucocytozoon caulleryi*, which represents the *Leucocytozoon* subgenus *Akiba*, was used as an outgroup following the phylogenetic reconstruction of haemosporidian parasites based on complete mitochondrial genomes (Pacheco et al. 2018). As an optimal model of molecular evolution, TIM2 model with gamma-distributed rate variation across sites (G) was selected based on the Bayesian Information Criterion (BIC) with jModelTest 2 0.1.11 (Darriba et al. 2012). However, because MrBayes 3.2.7 (Ronquist et al. 2012), which was used to reconstruct the phylogenetic tree, does not support TIM2 model, a general time reversible (GTR) model + G, which was the second best model selected by jModelTest 2, was used instead (see also Lecocq et al. 2013). Two separate runs of 10 million generations, each consisting of Metropolic-coupled MCMC with 4 chains (1 cold and 3 heated), with a sampling frequency of every 1000th generation were performed. The convergence of MCMC analyses and burn-in were assessed using Tracer 1.7.2 (Rambaut et al. 2018). The majority consensus tree was constructed based on the total number of 15,002 trees, which corresponds to burn-in of 25%. The phylogenetic tree was visualised and edited using FigTree 1.4.4. (available at http://tree.bio.ed.ac.uk/software/figtree/) with final editing performed in Inkscape 1.1.1. (available at https://inkscape.org).

The genetic distances between *Leucocytozoon* sequences were calculated in MEGA X using a Jukes-Cantor model of substitution with all substitutions weighted equally (Kumar et al. 2018).

### Statistical analyses

95% confidence intervals (95% CIs) for prevalence were calculated using the Sterne’s method (Klaschka and Reiczigel 2021). Differences in the proportion of infected birds between Whiskered and Black Terns and between populations of Whiskered Terns were tested with an unconditional exact test which is more sensitive than Fisher’s exact test in detecting differences, especially when sample sizes are small (*n*_1_, *n*_2_ < 100) (Reiczigel et al. 2019). In the analysis of an inter-specific difference in infection rates, only the population of Whiskered Terns in Ukraine was used to avoid potential bias which may arise in geographically distant populations due to e.g. differences in the abundance and composition of the vector community. The analyses were performed with Quantitative Parasitology (QPweb) 1.0.15 software available at https://www2.univet.hu/qpweb/qp10/index.php (Reiczigel et al. 2019).

## Results

### Prevalence of haemosporidian infections

Whiskered and Black Terns were infected with haemosporidians only sporadically. In total, six out of 214 samples with good quality DNA (as confirmed by amplification of DNA fragments from host sex chromosomes) were scored as containing haemosporidian parasites using multiplex PCR. In two of these samples—one containing coinfection of *Plasmodium* and *Leucocytozoon* and one with *Plasmodium* infection as assessed based on the size of fragments amplified with the multiplex PCR—the nested PCR either failed to amplify the *Plasmodium* sequence or the primer pair specific for *Plasmodium*/*Haemoproteus* amplified the product from the *Leucocytozoon* parasite (see Methods for details). Because of that we could neither verify the genus nor identify the lineage of *Plasmodium* parasites in these individuals. The lack of sequencing data also precluded verification of whether these *Plasmodium* infections were single or mixed. However, given a very low prevalence of haemosporidian parasites in screened marsh terns, we treated these *Plasmodium* infections as single. Only parasites from the genera *Plasmodium* and *Leucocytozoon* were detected. All infected terns except for one carried a single infection. Only individual Whiskered Tern from south-central Poland was infected both with *Plasmodium* and *Leucocytozoon*. Among five sequenced amplicons, all infections were single. At the population level, the overall haemosporidian prevalence (pooled data from different years) ranged from 0.034 (95% CI: 0.009 – 0.096) in south-central Poland to 0.022 (95% CI: 0.001 – 0.118) in north-central Ukraine in the Whiskered Tern and it was 0.024 (95% CI: 0.004 – 0.084) in the Black Tern. Whiskered and Black Terns did not differ in rates of haemosporidian infections (unconditional exact test, *p* > 0.30). There was also no difference in infection rates between the two populations of the Whiskered Tern (*p* > 0.30). No differences in prevalence were also found when *Plasmodium* and *Leucocytozoon* infections were analysed separately between tern species and populations of the Whiskered Tern (all *p* > 0.29).

Because only very few birds from each population were infected, we did not test for the effect of sex on the probability of infection. The prevalence of infection in females and males in each population was as follows: 0.025 (95% CI: 0.001 – 0.133) and 0.043 (95% CI: 0.008 – 0.146) in the Whiskered Tern in south-central Poland, 0.034 (95% CI: 0.002 – 0.169) and 0.000 (95% CI: 0.000 – 0.208) in the Whiskered Tern in north-central Ukraine, and 0.031 (95% CI: 0.002 – 0.166) and 0.020 (95% CI: 0.001 – 0.106) in the Black Tern.

### Lineage diversity and phylogenetic analyses

Among five amplicons produced with the nested PCR, we identified three haemosporidian lineages, all previously described: SGS1 representing *Plasmodium relictum* and CIAE02 and LARCAC02 representing *Leucocytzoon* spp. Host species from closely located populations did not differ in the number of parasite lineages, although they carried different lineages: Whiskered Terns—SGS1 and Black Terns—CIAE02. Whiskered Terns from south-central Poland carried more lineages than birds from the population in north-central Ukraine. Specifically, they carried in total at least three lineages: SGS1 (registered in both populations), LARCAC02, and two unidentified *Plasmodium* parasites (Table 2).

**Table 2:** Lineages of haemosporidian parasites detected in Whiskered and Black Terns at sampling locations in south-central Poland and north-central Ukraine. GenBank accession no: SGS1 – AF495571, LARCAC02 – KP271931, and CIAE02 – EF607287

Host species	Population location (sample size)	Parasite lineage (if identified) and number of infected individuals (in parentheses)	Morphospecies
Whiskered Tern	South-central Poland (*n* = 87)	SGS1 (1) Unidentified (2) LARCAC02 (1)	*Plasmodium relictum* *Plasmodium* spp. *Leucocytozoon* spp.
	North-central Ukraine (*n* = 45)	SGS1 (1)	*Plasmodium relictum*
Black Tern	North-central Ukraine (*n* = 82)	CIAE02 (2)	*Leucocytozoon* spp.

**Fig. 2:**
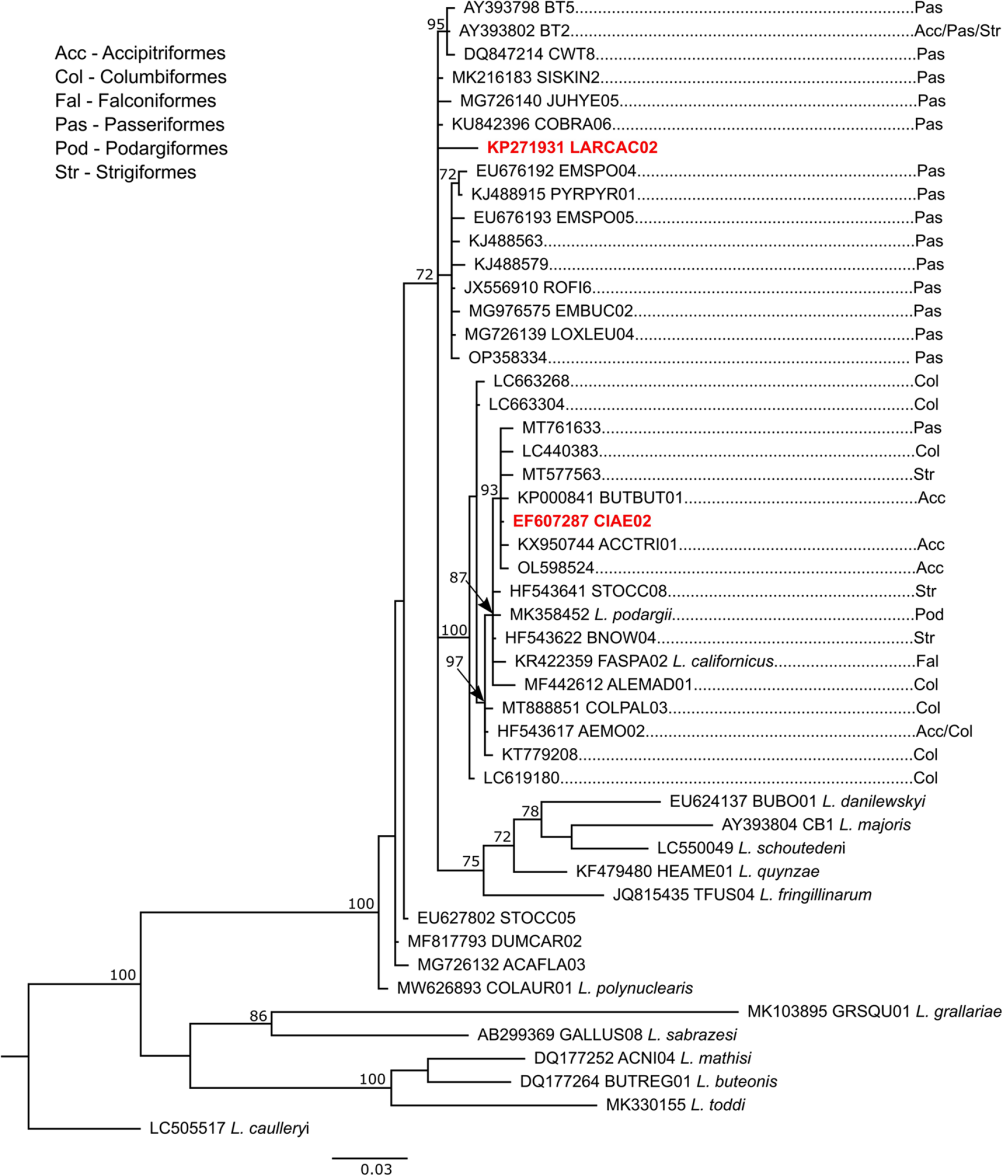
Phylogeny of selected *Leucocytozoon* parasites (closely rated to lineages CIAE02 and LARCAC02 and sequences matched to morphospecies) based on a 476-bp long fragment of the cytochrome *b* gene. The tree was reconstructed using Bayesian inference with *Leucocytozoon caulleryi* as an outgroup. Numbers associated with nodes represent posterior probabilities (with support over 70%). GenBank accession number, the MalAvi lineage name (if present), the name of the morphospecies (if identified), and known hosts of parasites closely related to CIAE02 and LARCAC02 are presented. The scale bar represents genetic distance

Phylogenetic analysis of *Leucocytozoon* lineages showed that the CIAE02 lineage is grouped in a highly supported clade with two morphospecies: *L. podagrii* and *L. californicus*, while LARCAC02 was not grouped with any morphospecies (Fig. 2). Genetic distances between CIAE02 and morphospecies were 0.42% in the case of *L. podagrii* and 0.63% in the case of *L. californicus*. The most closely related lineage to LARCAC02 – SISKIN2 showed a divergence of 1.91%. Lineages closely related to CIAE02 occur mostly in birds of prey and pigeons and doves, while all lineages most closely related to LARCAC02 have been registered only in passerines except for the BT2 lineage, which has been additionally reported in birds from orders Accipitriformes and Strigiformes (Fig. 2).

## Discussion

Two species of marsh terns screened in this study for the presence of haemosporidian parasites were infected only sporadically. In each of the three study populations, the infection rate did not exceed 3.4%. Such low infection rates are in accordance with findings in other terns, which have been shown to be either infected sporadically or to be free of haemoparasites (Table 1 and references therein). In contrast to the expectation of inter-specific differences in infection rates and/or the diversity of parasite communities, we found neither higher infection rates nor more diverse parasite communities in Whiskered than Black Terns in closely located populations. We also show that Whiskered Terns from two geographically isolated populations did not differ in infection rates, although birds from these populations carried a different number of lineages.

Haemosporidian infection rates in birds may be potentially driven by the host, vector, or parasite traits (Ferraguti et al. 2018; Eastwood et al. 2019; Ellis et al. 2020). In the study species of marsh terns, the low infection rates most probably are driven by vector exposure, which may be mediated through vector abundance, vector habitat preference, or host preference. Although in general water reservoirs such as fish ponds or dam reservoirs may be associated with a high abundance of dipterans such as mosquitoes and blackflies (Radrova et al. 2013; Zagalska-Neubauer and Bensch 2016), their distribution across these habitats may be non-uniform because of the small-scale variation in a physical environment (Bidlingmayer 1985). For example, mosquito host-seeking flight activity is being reduced with increasing wind velocity (Hoffmann and Miller 2003), and open areas should be associated with higher wind velocities. At our study sites, Whiskered and Black Terns nest on floating vegetation in some distance from the shores (Whiskered Terns: usually at least 40 m in south-central Poland and ca 2–4 km in north-central Ukraine, Black Terns: 60–120 m; N. Atamas, M. Ledwoń, personal observations). Nest platforms are also heavily used by birds of both species for roosting and loafing (Cramp 1985). Consequently, Whiskered and Black Terns may be exposed to vectors only on a limited scale leading to reduced transmission rates of haemosporidians. The possibility of microhabitat-related variation in vector exposure as a driver of variation in haemosporidian prevalence at closely located sampling sites have been, for example, suggested for passerines breeding in Arctic tundra (Bennett et al. 1992). The species nesting on the ground in more open forest-tundra habitat was found to be much less prevalently infected than the species nesting primarily in black spruce (*Picea mariana*) stands. Lothrop and Reisen (2001) showed that the abundance of host-seeking females of *Culex tarsalis* was much higher in microhabitats with elevated vegetation than in open microhabitats, such as sandbars and snags over water. Although we could not assess the exposure of marsh terns to blood-sucking dipterans at their nesting sites because no vector data were collected, we did observe vector activity primarily in the vicinity of shores. The expected general low abundance of host-seeking dipterans over open water surface may also explain the lack of an inter-population difference in the prevalence in the Whiskered Tern. Differences in haemosporidian prevalence among geographically distinct populations of the same species are commonly observed in avian hosts associated with other habitat types, e.g. forest (Dubiec et al. 2018). The possibility that vector exposure rather than high resistance drives low infection rates in Whiskered and Black Terns may be further supported by the observation in other larids that high exposure to vectors is associated with high infection rates. For example, in a mixed colony of two gull species sampled during breeding seasons with a high abundance of blackflies, infection rates in adult birds reached almost 100% (Zagalska-Neubauer and Bensch 2016). Moreover, in the Inca Tern (*Larosterna inca*)—a species which typically occupies habitats which are probably free of vectors: inshore “guano islands” along rocky coasts of west South America—infection rate with *Plasmodium* reached nearly 35% among birds within a zoological collection located outside of the species distribution range (Spottiswoode et al. 2020). Importantly, this relatively high infection rate occurred despite taken preventive measures. These included relocation of young individuals (shortly before fledging) indoors to decrease exposure to mosquitoes and administering antiprotozoal medication for 3 months before the birds were released again outdoors.

As of 23 March 2023, 20 haemosporidian lineages (eight *Plasmodium*, ten *Haemoproteus*, and two *Leucocytozoon*) have been registered in wild larids including three lineages in wild injured individuals sampled in rescued centres (Bensch et al. 2009; Capasso et al. 2023; Vanstreels et al. 2023). CIAE02 and LARCAC02 are the only *Leucocytozoon* lineages reported in this family so far. Two lineages registered in this study—SGS1 in the Whiskered Tern and CIAE02 in the Black Tern—are generalist parasites as they have been reported from a broad range of hosts and have a broad geographic distribution. SGS1 has been registered on all continents except for Antarctica in146 host species from 36 families representing 12 orders (Anseriformes, Charadriiformes, Ciconiiformes, Columbiformes, Galliformes, Gruiformes, Passeriformes, Phoenicopteriformes, Procellariformes, Sphenisciformes, Strigiformes, Trochiliformes) and CIAE02—in Asia, Europe, and Africa in 31 species from 13 families representing ten orders (Accipitriformes, Charadriiformes, Ciconiiformes, Coraciiformes, Cuculiformes, Falconiformes, Gruiformes, Phoenicopteriformes, Piciformes, Strigiformes) (the MalAvi database, Bensch et al. 2009). Both lineages have also been registered in wild populations of three larid species: three gull species sampled during the breeding season in Poland (the Caspian Gull, the Herring Gull, and the Black-headed Gull) and one gull species (the Caspian Gull) sampled on breeding grounds in Mongolia (Zagalska-Neubauer and Bensch 2016; Seimon et al. 2016; Włodarczyk et al. 2022). In all larid hosts, infections with each of the two lineages occurred at low rates, not exceeding 10%. In contrast to SGS1 and CIAE02, LARCAC02 has a narrow host as well as geographic range, which suggests that this is a specialist parasite. Apart from a single Whiskered Tern in southern Poland reported in this study, it has been reported to date only in three other larids: the Herring Gull, the Caspian Gull (and their hybrids), and the Common Tern sampled at two locations in central Poland (Zagalska-Neubauer and Bensch 2016; Włodarczyk et al. 2022). Zagalska-Neubauer and Bensch (2016) detected LARCAC02 as a dominant haemosporidian parasite in a mixed-species gull colony at the Włocławek reservoir located on the Vistula River. In a set of 56 gulls, either Herring Gulls, Caspian Gulls, or their hybrids, 96% of individuals carried LARCAC02 infection. Some gull species may, therefore, be a reservoir of this lineage. Interestingly, Włodarczyk et al. (2022) did not detect LARCAC02 in 140 Black-headed Gulls sampled in seven colonies located across Poland. In the Common and Whiskered Terns, LARCAC02 occurred sporadically—in both cases with the infection rate below 1.5% (this study, Włodarczyk et al. 2022). Infections of LARCAC02 in the Common and Whiskered Terns most probably represent spillover of parasites from species that are main hosts for this lineage, e.g. Caspian and Herring Gulls. Although Whiskered Terns and the two gull species do not form mixed colonies, the nearest studied colony of Whiskered Terns on a carp pond complex in south-central Poland was located ca 3 km from a colony of Caspian Gulls and both species may forage at the same locations (M. Ledwoń, personal observation). Importantly, because neither in this nor Włodarczyk et al. (2022) study blood smears were collected, and therefore, it was not possible to confirm the presence of gametocytes in the blood, it may not be excluded that terns represent dead-end host, i.e. species in which parasites may not complete its life cycle (Valkiūnas et al. 2009).

Among three detected haemosporidian parasites, only vectors of SGS1 have been identified to date. Based on the occurrence of this parasite DNA in the head and thorax of mosquitoes, which may suggest the presence of the infective stage of the parasite—sporozoites, *Cx. pipiens*, *Cx. modestus*, *Cx. theileri*, *Cx. perexiguus*, and *Culiseta annulata* may act as vectors (the MalAvi database, Bensch et al. 2009). Out of these five species, only *Cx. pipiens* and *Cx. modestus* were confirmed as competent vectors of SGS1 based on the microscopic detection of sporozoites, which is necessary to verify sporogony (Kim and Tsuda 2015; Dimitrov et al. 2023). The transmission of SGS1 has been confirmed on all continents except for Antarctica (the MalAvi database, Bensch et al. 2009), so Whiskered Terns breeding in Poland and Ukraine may get infected with this parasite either on breeding grounds, wintering grounds, or during migration at stopover sites. *Leucocytozoon* parasites are transmitted either by blackflies (subgenus *Leucocytozoon*) or *Culicoides* midges (subgenus *Akiba* represented by a single species *Leucocytozoon caulleryi*) (Santiago-Alarcon et al. 2012). Because very high rates of *Leucocytozoon* infections in gulls in the Włocławek reservoir coincided with a massive occurrence of blackflies (Zagalska-Neubauer and Bensch 2016), infections were probably mostly transmitted locally. This should be especially true for LARCAC02 because its distribution has been restricted up to date to three sampling sites (including this study) in Poland. CIAE02 on the other hand has a broad transmission range spreading over Europe, Asia, and Africa (the MalAvi database, Bensch et al. 2009). Sampling of adult blackflies in the Włocławek reservoir area over an 8-year long period including two seasons when Caspian and Herring Gulls were sampled showed that the community of simuliids was composed of three species: *Simulium maculatum*, *S. erythrocephalum*, and *S. pusillum* with prevalences of 55%, 42%, and 3%, respectively (K. Szpila and T. Kakareko personal communication, as cited in Zagalska-Neubauer and Bensch 2016). Out of these three species, only *S. maculatum* is classified, based on the claw structure adapted for grasping the feathers and the presence of *Leucocytozoon* DNA in the representative of the species (blackflies may get infected with *Leucocytozoon* parasites only when taking a blood meal from an infected bird), as ornithophilic (Malmqvist et al. 2007; Žiegytė and Bernotienė 2022), making it the most probable vector of LARCAC02 and CIAE02 in the Włocławek reservoir. The possibility that a single simuliid species may transmit different *Leucocytozoon* species has been shown experimentally (Desser and Bennett 1993). Both *Leucocytozoon* lineages may be potentially vectored also by other ornithophilic blackflies, because *Leucocytozoon* parasites, either identified to morphospecies or genetic lineage level, are commonly recorded in more than one simuliid species (Santiago-Alarcon et al. 2012; Chakarov et al. 2020). According to the most recent revision of the taxonomic and geographic inventory of world blackflies, the occurrence of 52 species has been confirmed in Poland (Adler 2022).

The phylogenetic reconstruction showed that LARCAC02 did not form a well-supported clade with any of the morphospecies, while CIAE02 grouped in a highly supported clade with two morphospecies: *L. californicus* and *L. podagrii*. *L. californicus* was first described in the American Kestrel (*Falco sparverius*) in North America (Walther et al. 2016), and *L. podagrii*—in the Tawny Frogmouth (*Podargus strigoides*) in Australia (Adlard et al. 2002). The only lineage linked so far to *L. californicus* FASPA02 has been recently detected in the falconiform raptor in Italy, confirming that this parasite occurs in Europe (Nardoni et al. 2020). To verify whether CIAE02 is a genetic variant of *L. californicus*, the microscopic analysis of gametocytes is necessary (Valkiūnas 2005), because even lineages with a very small genetic distance, corresponding to a difference of one nucleotide over an approximately 480-bp long fragment of the cytochrome *b* gene, may represent different morphospecies (Levin et al. 2012).

Summing up, our study is the first one exploring molecularly in a large sample the occurrence of haemosporidian parasites in two species of terns representing the genus *Chlidonias*. To get a better understanding of infection patterns with haemosporidians from genera *Plasmodium*, *Haemoproteus*, and *Leucocytozoon* in this group, birds should be also sampled on wintering grounds. Moreover, to elucidate to what degree vectors shape low infection rates in these birds, sampling of vectors is required.

## Data Availability

The dataset associated with this study is available in the Open Science Framework repository: 10.17605/OSF.IO/YN5TP.
